# Outbreak of *Salmonella* Newport Infections with Decreased Susceptibility to Azithromycin Linked to Beef Obtained in the United States and Soft Cheese Obtained in Mexico — United States, 2018–2019

**DOI:** 10.15585/mmwr.mm6833a1

**Published:** 2019-08-23

**Authors:** Ian D. Plumb, Colin A. Schwensohn, Laura Gieraltowski, Selam Tecle, Zachary D. Schneider, Jennifer Freiman, Andrea Cote, Douglas Noveroske, Jonathan Kolsin, Joshua Brandenburg, Jessica C. Chen, Kaitlin A. Tagg, Porscha Bumpus White, Hazel J. Shah, Louise K. Francois Watkins, Matthew E. Wise, Cindy R. Friedman

**Affiliations:** ^1^Division of Foodborne, Waterborne, and Environmental Diseases, National Center for Emerging and Zoonotic Infectious Diseases, CDC; ^2^California Department of Public Health; ^3^Oak Ridge Institute for Science and Education, Tennessee; ^4^Food Safety and Inspection Service, U.S. Department of Agriculture, Washington, DC; ^5^Texas Department of State Health Services; ^6^Weems Design Studio Inc., Suwanee, Georgia.

In September 2018, CDC identified *Salmonella enterica* serotype Newport (Newport) infections that were multidrug resistant (MDR), with decreased susceptibility to azithromycin, a recommended oral treatment agent. Until 2017, decreased susceptibility to azithromycin had occurred in fewer than 0.5% of *Salmonella* isolates from U.S. residents. This report summarizes the investigation of a multistate MDR *Salmonella* outbreak conducted by CDC, state and local health departments, and the U.S. Department of Agriculture’s Food Safety and Inspection Service. During June 2018–March 2019, 255 cases of infection with the outbreak strain were identified in 32 states; 43% of patients (89 of 206 with information on travel) reported recent travel to Mexico. Infections were linked to consumption of soft cheese obtained in Mexico and beef obtained in the United States. Consumers should avoid eating soft cheese that could be made from unpasteurized milk, regardless of the source of the cheese. When preparing beef, a food thermometer should be used to ensure that appropriate cooking temperatures are reached. When antibiotic treatment is needed for a patient, clinicians should choose antibiotics based on susceptibility testing wherever possible.

## Epidemiologic Investigation

In 2018, during an investigation of antibiotic-susceptible Newport infections that led to a U.S. ground beef recall (*1*), a genetically distinct group of MDR Newport isolates was identified. Isolates were classified as the outbreak strain if they fell within the MDR clade (0–11 alleles by core genome multilocus sequence typing [cgMLST]); isolates were identified using PulseNet, the national subtyping network for foodborne bacterial disease surveillance. A case was defined as isolation of the outbreak strain from a patient during June 2018–March 2019. After interviews conducted by state and local health departments, some patients were reinterviewed using a standardized hypothesis-generating questionnaire or supplementary questionnaires that included questions about travel and antibiotic treatment. Food exposures were reported for the 7 days before illness onset. Exposures among patients who did not travel internationally were compared with those expected among a nationally representative sample of healthy persons included in the U.S. Foodborne Diseases Active Surveillance Network population survey (2006–2007) (*2*), after stratification by sex and ethnicity.

During June 2018–March 2019, 255 cases were identified in 32 U.S. states (Figure). Overall, 29% (60/209) of patients for whom information was available were hospitalized, 6% (4/70) were admitted to an intensive care unit, 4% (10/255) had *Salmonella* bacteremia, and two died. The median patient age was 36 years (range = <1–90 years), 58% (145/250) were female, and 65% (143/221) were Hispanic. Overall, 43% (89/206) with information on travel reported visiting Mexico in the 7 days preceding illness onset. Travelers to Mexico mostly reported visiting friends or family (67%, 24/36) and collectively reported visiting 16 of the 32 states within Mexico. Patients who did not visit Mexico were residents of 26 U.S. states.

**FIGURE:**
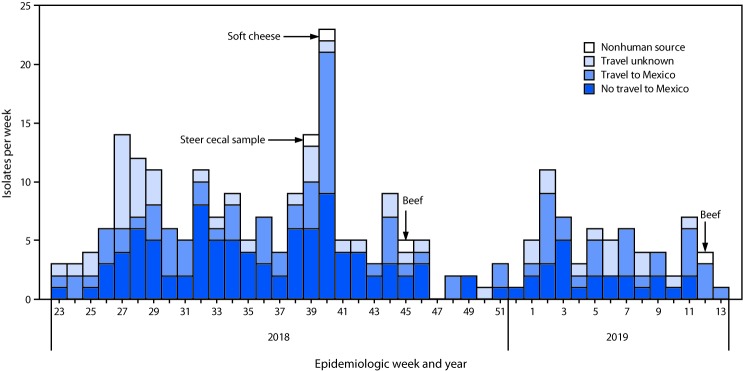
Identification of isolates of the outbreak strain of *Salmonella enterica* serotype Newport from infected patients (N = 255), by travel status,* and from nonhuman sources^†^ (n = 4), by epidemiologic week and year — United States, June 2018–March 2019 * Defined as reported travel within 7 days before illness onset. ^†^ Cecal sample and beef samples obtained in the United States; sample of cheese obtained in Mexico by a patient infected with the outbreak strain who consumed this cheese.

Among patients who traveled to Mexico with information on food consumption, 87% (41/47) reported eating beef, and 63% (29/46) reported eating soft cheese; among those, 79% (23/29) recalled obtaining the cheese in Mexico (Table 1). Of several types of artisanal cheese reported, the most frequently recalled cheese was queso fresco, a cheese that is typically made with raw, unpasteurized milk from cows or goats (*3*). Among patients who did not travel to Mexico, 29% (20/70) reported eating Mexican-style soft cheese, and 93% (68/73) reported eating beef (Table 1). The percentage who ate Mexican-style soft cheese was similar to the percentage in the nationally representative sample of healthy persons (p-value = 0.54), whereas the percentage who ate beef was higher than that among healthy persons (p<0.01).

**TABLE 1:** Consumption of beef or Mexican-style soft cheese within 7 days of illness onset among patients (N = 255) with the outbreak strain of *Salmonella enterica* serotype Newport — United States, June 2018–January 2019

Reported exposure within 7 days of illness onset	No./No. with available information (%)	Patients with known travel status (n = 206)	
		No. who visited Mexico* (%)	No. who did not visit Mexico* (%)
**Any beef**			
No	11/121 (9)	6/47 (13)	5/73 (7)
Yes	110/121 (91)	41/47 (87)	68/73 (93)
**Source of beef**			
United States	55/110 (50)	7/41 (17)	48/68 (71)
Mexico	18/110 (16)^†^	17/41 (42)	1/68 (1)^†^
Unknown	38/110 (35)	17/41 (42)	20/68 (29)
**Type of beef**			
Other	24/110 (22)	12/41 (29)	12/68 (18)
Ground	60/110 (55)	18/41 (44)	41/68 (60)
Unknown	26/110 (24)	11/41 (27)	15/68 (22)
**Any Mexican-style cheese**			
No	68/118 (58)	17/46 (37)	50/70 (71)
Yes	50/118 (42)	29/46 (63)	20/70 (29)
**Source of Mexican-style cheese**			
United States	15/50 (30)^§^	3/29 (10)^§^	12/20 (60)
Mexico	29/50 (58)	23/29 (79)	5/20 (25)
Unknown	7/50 (14)	4/29 (14)	3/20 (15)

* Of patients with known travel status, 89 had visited Mexico, and 117 had not visited Mexico.

^†^ One patient who did not travel to Mexico reported eating beef obtained in Mexico in addition to beef obtained in the United States

^§^ One patient who traveled to Mexico reported eating Mexican-style soft cheese obtained in the United States and also Mexican-style soft cheese obtained in Mexico.

## Product and Animal Testing

In September 2018, the outbreak strain was detected in a cecal sample from a steer collected at a slaughter and processing plant in Texas as part of National Antimicrobial Resistance Monitoring System (NARMS) surveillance (Figure). In October 2018, the outbreak strain was detected in a mixture of queso fresco and Oaxaca soft cheese purchased in a market in Tijuana, Mexico. The cheese had been brought into the United States by a patient who became ill with a strain that was indistinguishable (0 allele difference) from the strain isolated from the cheese. The outbreak strain was detected in beef samples collected in November 2018 and March 2019 at two Texas slaughter and processing facilities. Isolates from the Mexican cheese, the steer cecum, and beef differed by 0–2 alleles from one another and by a minimum of 0–1 alleles from patient isolates (Table 2). Review of patient information did not identify any common suppliers of contaminated beef or cheese.

**TABLE 2:** Characteristics of four isolates from nonhuman sources closely related to the outbreak strain of *Salmonella enterica* serotype Newport — United States, June 2018–January 2019

Isolate no.*	Isolation date	Source of isolate	Notes on source	Median no. of alleles different from patient isolates (range)
1	9/6/2018	Steer (cecum)	Texas slaughter and processing facility	3 (1–7)
2	10/05/2018	Cheese^†^	Mixture of Oaxaca and queso fresco	2 (0–5)
3	11/09/2018	Beef trim	Texas slaughter and processing facility	4 (0–8)
4	3/18/2019	Boneless beef	Texas slaughter and processing facility	3 (1–7)

* Isolates were within 0–2 alleles of each other by core genome multilocus sequence typing.

^†^ Obtained from the home of a patient who consumed some of it within 7 days before illness onset; the cheese was purchased from a market in Tijuana, Mexico.

## Antibiotic Resistance

Antibiotic resistance was predicted using whole genome sequencing and confirmed in a subset of isolates by antimicrobial susceptibility testing using broth microdilution; decreased susceptibility to azithromycin was defined as minimum inhibitory concentration ≥32 *μ*g/mL (*4*). Of 252 isolates with resistance information, 226 (90%) had predicted resistance to trimethoprim-sulfamethoxazole, tetracycline, and chloramphenicol, and decreased susceptibility to azithromycin. In 143 (57%) isolates, there was additional predicted resistance to ampicillin and streptomycin, and nonsusceptibility to ciprofloxacin (defined as minimum inhibitory concentration ≥0.12 *μ*g/mL) (*4*). All resistance genes were located on an IncR plasmid. Among patients with treatment information, 65/87 (75%) received antibiotic therapy, and 28/86 (33%) received an antibiotic to which the outbreak strain was resistant or showed decreased susceptibility.

## Discussion

This investigation identified an MDR strain of *Salmonella* Newport with decreased susceptibility to azithromycin and nonsusceptibility to ciprofloxacin, two oral agents recommended for treatment of *Salmonella* infections. The presence of resistance genes on a plasmid is concerning because of the potential for spread to other bacteria (*5*). The outbreak strain appears to have emerged recently because Newport with decreased susceptibility to azithromycin was not detected in animal, retail meat, or human isolates in NARMS surveillance before 2016 (*4*). During 2016–2017, two smaller multistate clusters of MDR Newport infections with decreased susceptibility to azithromycin were investigated among U.S. residents; isolates were within 11 alleles of the current outbreak isolates. No source of the infections was identified, but a high percentage of patients reported recent travel to Mexico (Division of Foodborne, Waterborne, and Environmental Diseases, National Center for Emerging and Zoonotic Infectious Diseases, CDC, unpublished data, 2019). Routine monitoring in 2016 detected an isolate from a sample of beef imported from Mexico that was indistinguishable (0 allele difference) from the outbreak strain isolated from cheese in 2018.

In this MDR outbreak, consumption of cheese and consumption of beef were both associated with illness, indicating that dairy cattle were a likely source of these infections. The detection of the outbreak strain in cheese purchased in Mexico and the high percentage of travelers to Mexico who reported eating Mexican-style soft cheese suggest that soft cheese from Mexico was a source of infection. Mexican-style soft cheese has been previously identified as a source of other *Salmonella* outbreaks (*6*). The reported consumption of queso fresco, travel to various regions in Mexico, and detection of indistinguishable Newport strains in beef and cheese suggest that contamination of soft cheese resulted from carriage by cattle rather than poor hygiene during cheese production. Dairy cattle often are used as a source of ground beef and have been implicated in previous MDR Newport outbreaks (*5*).

Among patients who did not travel to Mexico, beef was identified as a source of infection by the close genetic relatedness between isolates from patients and beef samples, and from the higher percentage of patients who ate beef compared with the percentage of healthy persons who ate beef. It is also possible that beef was a source of infection among some travelers to Mexico; nearly 90% of them also reported eating beef, and in 2016 the outbreak strain was detected in beef imported from Mexico.

The genetic similarity between isolates from beef in Mexico, beef in the United States, and a steer in the United States strongly suggests that the outbreak strain is present in cattle in both countries. Because use of antibiotics in livestock can cause selection of resistant strains (*7*), the reported 41% rise in macrolide use in U.S. cattle from 2016 to 2017 (*8*) might have accelerated carriage of the outbreak strain among U.S. cattle. Avoiding the unnecessary use of antibiotics in cattle, especially those that are important for the treatment of human infections, could help prevent the spread of MDR Newport with decreased susceptibility to azithromycin. Further investigation is warranted to determine the prevalence of Newport with decreased susceptibility to azithromycin in U.S. and Mexican cattle, and to identify measures to prevent transmission among cattle.

Whole genome sequencing was valuable in linking human infections to food sources, distinguishing the MDR outbreak strain from an antibiotic-susceptible strain causing a simultaneous outbreak, and predicting antibiotic resistance. In this outbreak, one in three patients received an antibiotic that was likely to have been ineffective. Clinicians should limit use of antibiotics for patients with an acute diarrheal illness to those with clinical indications (*9*), and antibiotic selection should be based on susceptibility results whenever possible. For empiric treatment of patients with suspected Newport with decreased susceptibility to azithromycin, ceftriaxone or alternative agents should be considered. To prevent infection, consumers should avoid eating soft cheese that could be made with unpasteurized milk, and when preparing beef they should use a thermometer to ensure appropriate cooking temperatures are reached: 145°F (62.8°C) for steaks and roasts followed by a 3-minute rest time, and 160°F (71.1°C) for ground beef or hamburgers (*10*).

**Summary**

**What is already known about this topic?**

Decreased susceptibility to azithromycin is rare among *Salmonella* serotypes that cause human infections in the United States. If antibiotic treatment is indicated, azithromycin is recommended as an oral therapy.

**What is added by this report?**

During June 2018–March 2019, an outbreak caused by multidrug-resistant *Salmonella* Newport with decreased susceptibility to azithromycin led to 255 infections and 60 hospitalizations. Infections were linked to Mexican-style soft cheese obtained in Mexico and beef obtained in the United States.

**What are the implications for public health practice?**

Whole genome sequencing can be used in *Salmonella* outbreak investigations for rapid prediction of antimicrobial resistance and can link cases to each other and to possible sources of infection.
