# *Erratum:* Vol. 68, No. 33

**DOI:** 10.15585/mmwr.mm6840a6

**Published:** 2019-10-11

In the report “Outbreak of *Salmonella* Newport Infections with Decreased Susceptibility to Azithromycin Linked to Beef Obtained in the United States and Soft Cheese Obtained in Mexico — United States, 2018–2019,” on page 716, in the first full paragraph, the second sentence should read “Because use of antibiotics in livestock can cause selection of resistant strains (*7*), the reported 41% rise in macrolide **sales for use** in U.S. cattle from 2016 to 2017 (*8*) might have accelerated carriage of the outbreak strain among U.S. cattle.”

In addition, reference 8 should have read as follows: “8. Food and Drug Administration. 2017 summary report on antimicrobials sold or distributed for use in food-producing animals. (**Page 24, 5b)**. Silver Spring, MD: US Department of Health and Human Services, Food and Drug Administration; 2018. https://www.fda.gov/media/119332/download”

